# Pharmacokinetic and Metabolomic Studies with BIO 300, a Nanosuspension of Genistein, in a Nonhuman Primate Model

**DOI:** 10.3390/ijms20051231

**Published:** 2019-03-12

**Authors:** Amrita K. Cheema, Khyati Y. Mehta, Paola T. Santiago, Oluseyi O. Fatanmi, Michael D. Kaytor, Vijay K. Singh

**Affiliations:** 1 Department of Oncology, Lombardi Comprehensive Cancer Center, Georgetown University Medical Center, Washington, DC 20057, USA; amrita.cheema@georgetown.com (A.K.C.); kym8@georgetown.com (K.Y.M.); 2 Department of Biochemistry, Molecular and Cellular Biology, Georgetown University Medical Center, Washington, DC 20057, USA; 3 Department of Pharmacology and Molecular Therapeutics, F. Edward Hébert School of Medicine, USUHS, Bethesda, MD 20814, USA; paola.santiago.ctr@usuhs.edu (P.T.S.); oluseyi.fatanmi@usuhs.edu (O.O.F.); 4 Armed Forces Radiobiology Research Institute, USUHS, Bethesda, MD 20814, USA; 5 Humanetics Corporation, Edina, MN 55435, USA; mkaytor@humaneticscorp.com

**Keywords:** BIO 300, biomarkers, genistein, metabolomics, nonhuman primates, radiation countermeasure, serum

## Abstract

Genistein is a naturally occurring phytoestrogen isoflavone and is the active drug ingredient in BIO 300, a radiation countermeasure under advanced development for acute radiation syndrome (H-ARS) and for the delayed effects of acute radiation exposure (DEARE). Here we have assessed the pharmacokinetics (PK) and safety of BIO 300 in the nonhuman primate (NHP). In addition, we analyzed serum samples from animals receiving a single dose of BIO 300 for global metabolomic changes using ultra-performance liquid chromatography (UPLC) quadrupole time-of-flight mass spectrometry (QTOF-MS). We present a comparison of how either intramuscularly (*im*) or orally (*po*) administered BIO 300 changed the metabolomic profile. We observed transient alterations in phenylalanine, tyrosine, glycerophosphocholine, and glycerophosphoserine which reverted back to near-normal levels 7 days after drug administration. We found a significant overlap in the metabolite profile changes induced by each route of administration; with the *po* route showing fewer metabolic alterations. Taken together, our results suggest that the administration of BIO 300 results in metabolic shifts that could provide an overall advantage to combat radiation injury. This initial assessment also highlights the utility of metabolomics and lipidomics to determine the underlying physiological mechanisms involved in the radioprotective efficacy of BIO 300.

## 1. Introduction

Clinical preparedness for the global threat of a nuclear disaster is a critically important priority for all US agencies involved in domestic security and public health preparedness [1]. It is widely perceived that terrorist organizations have the capability to obtain or engineer an improvised nuclear device or other type of radiological dispersal device [2]. Radiation countermeasures that can reduce or eliminate the public health impact of radiation exposure are needed [3]. Only three drugs, Neupogen^®^ (granulocyte-colony stimulating factor, G-CSF), Neulasta^®^ (pegylated G-CSF), and Leukine^®^ (granulocyte-macrophage colony-stimulating factor, GM-CSF) have been approved by the US Food and Drug Administration (US FDA) as radiomitigators for the hematopoietic acute radiation syndrome (H-ARS) [4,5,6,7,8]. Administration of any of the above three drugs requires close patient monitoring by a highly skilled medical team that may not be available during a mass casualty event.

Humanetics Corporation is developing BIO 300 as a radiation countermeasure to prevent and mitigate the effects of ARS and the DEARE. The active drug ingredient in BIO 300 is unconjugated, synthetic genistein [5,7-dihydroxy-3-(4-hydroxyphenyl)-chromen-4-one, C_15_H_10_O_5_, 270.237 g/mol]. Genistein is a naturally occurring isoflavone found in soy that has been shown to have radioprotective and mitigating properties [9,10,11,12,13]. The commercial development of genistein has been limited by its physical properties. Genistein is poorly soluble in water and aqueous formulations and thus has low bioavailability [14]. To advance the pharmaceutical development of genistein Humanetics Corporation has developed a proprietary aqueous suspension of synthetic genistein nanoparticles (BIO 300). BIO 300 is manufactured using a wet-nanomilling process that reduces the mean genistein particle size to less than 200 nm. This process vastly improves the bioavailability of BIO 300, resulting in doses that are efficacious with either parenteral or *po* administration [15,16]. Long-term and accelerated stability studies have shown BIO 300 to be stable for up to 36 months when stored at ambient temperature (15–30 °C).

Genistein’s mechanism of action as a radioprotectant is not completely understood, but its purported mechanism of action is as a selective agonist of estrogen receptor beta (ERβ). The two estrogen-activated transcription factors (ERα and ERβ) act in an antagonistic manner. While ERα activation is attributed to cellular growth (ERα is a driver of 50–80% of breast cancers), ERβ functions as a negative feedback regulator, thus responsible for activating cell cycle checkpoints and repressing cell growth [17]. Genistein activation of ERβ has been reported to occur at nanomolar concentrations, and it has an IC_50_ of 8.4 nm [18]. Notably, the proliferative rate of cells is directly related to their radiosensitivity, thus it is hypothesized that genistein administration diminishes the growth rate of cells, which increases cellular radioresistance. When administered prophylactically, genistein protects against acute myeloid injury by mediating extended quiescence and reduced senescence of hematopoietic stem cells [10,12]. Genistein has been shown to arrest hematopoietic stem cells at the G2/M phase of the cell cycle, inducing a senescent state and thus, reducing the deleterious effects resulting from radiation exposure [10,12,19]. Upon release from cell cycle arrest, the stem cells are able to quickly repopulate the bone marrow. In addition to its effects on cell signaling, genistein’s antioxidant properties include the ability to directly scavenge reactive oxygen species (ROS) that are implicated in the formation of cellular oxidative damage including DNA double strand breaks [20,21]. A single dose of BIO 300 (200 mg/kg) administered subcutaneously (*sc*) or *im* 24 h prior to total-body irradiation (TBI) significantly improves survival in mice [16]. BIO 300 has been investigated as a mitigator of lethal radiation-induced pneumonitis/fibrosis in a well-established murine model of whole thorax lung injury (WTLI) [22,23,24]. BIO 300 significantly improved survival compared to untreated animals when initiated 24 h post-exposure (11 Gy, LD_50/180_ or 12.5 Gy, LD_90/180_) and continued once a day for 4–6 weeks.

Currently, we are studying BIO 300 in NHPs for its efficacy and mechanism of action against ionizing radiation-induced H-ARS and DEARE. BIO 300 is being developed following the US FDA Animal Rule as a radioprotector and radiomitigator for ARS/DEARE, which can be administered either prior to or after radiation exposure [25]. Here, we have studied BIO 300 pharmacokinetics (PK) and changes induced by BIO 300 in the metabolomic and lipidomic profiles as part of its safety and toxicity in a NHP model. We report BIO 300 PK and longitudinal changes in metabolic and lipidomic serum profiles of NHPs using a global metabolomics approach with an ultra-performance liquid chromatography (UPLC) quadrupole time-of-flight mass spectrometry (QTOF-MS) platform. Taken together, our results suggest that BIO 300 administration does not have adverse consequences on overall metabolism and is safe for use as a radiation countermeasure.

## 2. Results

### 2.1. Pharmacokinetics of BIO 300 Administered po or im

**Figure 1:**
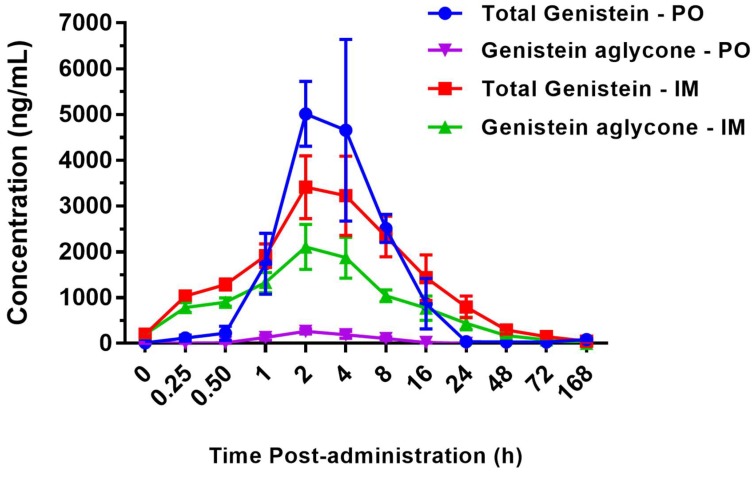
Pharmacokinetic analysis of BIO 300: Oral (*po*) vs intramuscular (*im*) administration. BIO 300 (50 mg/kg, *im* and 100 mg/kg, *po*) was administered to four nonhuman primates (NHPs). Blood samples were collected at various time points following BIO 300 administration. Total (glucuronidated genistein and genistein aglycone) and genistein aglycone concentrations were determined using a validated bioanalytical method.

Pharmacokinetic parameters were determined using serum levels of total and genistein aglycone quantified as described in Materials and Methods and the results are presented in Figure 1. The data presented indicate that the serum level of genistein aglycone (active form) is dependent on the route of BIO 300 administration. Animals receiving BIO 300 by *im* injection had higher serum concentration of genistein aglycone as compared to those that received the drug by the *po* route. Additionally, the serum concentrations of the drug were highest in a 2–8 h time window post-administration. Pharmacokinetic analyses of serum concentrations following *po* or *im* administration for both total and genistein aglycone are presented in Table 1 and Table 2. These tables display individual values obtained from each animal as well as average values for T_max_, C_max_, T_1/2_, AUC_0-48_, and AUC_0-**∞**_ for total and genistein aglycone when BIO 300 was administered through *im* or *po* route. 

### 2.2. Analysis for CBC and Vital Signs in NHP Administered BIO 300

Following BIO 300 administration through either the *im* or *po* route, complete blood counts (CBC) were analyzed starting 7 days prior to irradiation and continuing until 21 days post drug administration. During this period, vital signs of animals were also recorded. CBC analysis revealed similar hematological profiles after *im* and *po* administration of BIO 300. Graphs for CBC parameters are presented in Figures S1 and S2. Vital signs monitored throughout both studies included blood pressure, weight, percent change in weight, heart rate, and temperature and are presented in Figures S3 and S4.

### 2.3. Identification of Metabolite Signatures of BIO 300 Administration in NHPs

**Figure 2:**
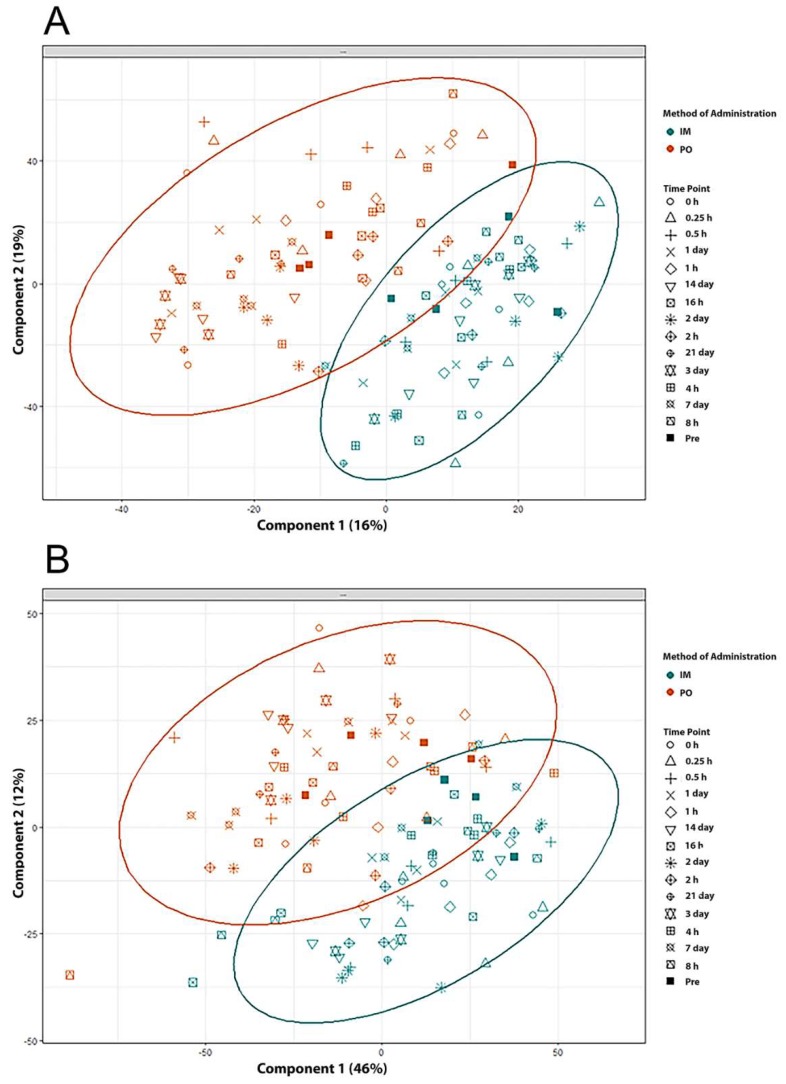
PLS-DA analysis to determine longitudinal changes in metabolomic profiles after BIO 300 administration by either the *im* or *po* routes, in ESI positive (panel **A**) and ESI negative (panel **B**) mode.

Untargeted metabolomic profiling was performed using 120 serum samples obtained from four NHPs. The serum samples were collected at various times post BIO 300 administration (either *im* or *po*). A total of 3702 features in the Electrospray Ionization (ESI) positive mode and 3206 features in the ESI negative mode were detected for metabolomics (Acquity BEH C18 column). Score plots (Figure 2, Panels A and B) in positive and negative electrospray (ESI) modes were used to evaluate group separation resulting from inherent differences in metabolite profiles of NHPs that received BIO 300 from the two different routes of administration in a time dependent manner. The coefficient of variation (CV) values of internal standards for quality control samples (QCS) as well as the base peak intensity chromatogram (BPI) overlays have been presented as Figure S5. 

**Figure 3:**
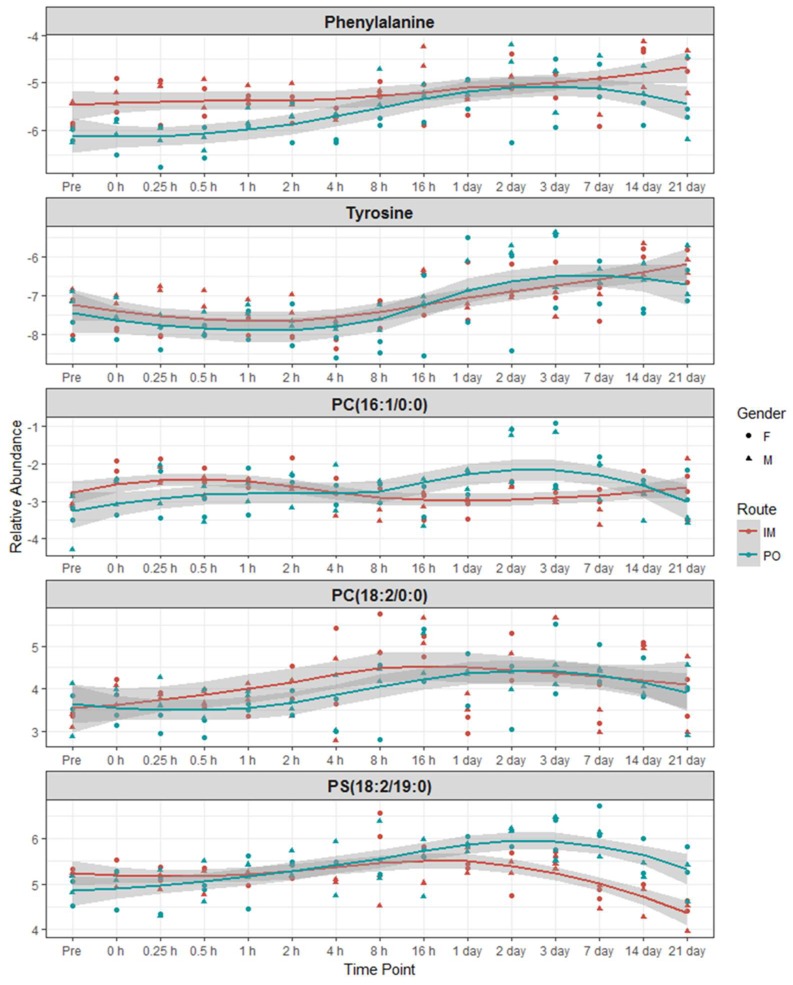
ANOVA analyses help delineate metabolites showing a common trend following each route of administration.

We aimed to determine if BIO 300 administration induced changes in longitudinal (time of BIO 300 administration to 21 days) and/or if overall metabolite profiles changed depending on the route of administration (*im* vs *po*). PLS-DA analysis comparing metabolic profiles of NHPs receiving BIO 300 by *im* vs *po* resulted in multi-class model including two components yielded *R*^2^ = 0.79 and *Q*^2^ = 0.73 in the positive mode, suggesting a modest separation between the two groups. Remarkably, there was a significant “within” group overlap between metabolomic profiles of NHPs for all the time points monitored pre- and post-administration of the drug. These results suggest that administration of BIO 300 induced modest metabolic changes. Since we observed a partial overlap between metabolic profiles in NHPs receiving BIO 300 by *im* vs *po* route of administration we sought to identify common metabolic changes. These analyses helped identify modest changes in serum levels of amino acids phenylalanine and tyrosine, glycerophosphocholine and glycerophosphoserine (Figure 3). Interestingly, maximum changes in the endogenous levels of these metabolites were observed between 3–7 days following a single BIO 300 administration with the serum levels reverting back to pre-administration levels by day 14, indicating that the metabolic changes observed were transient in nature.

**Figure 4:**
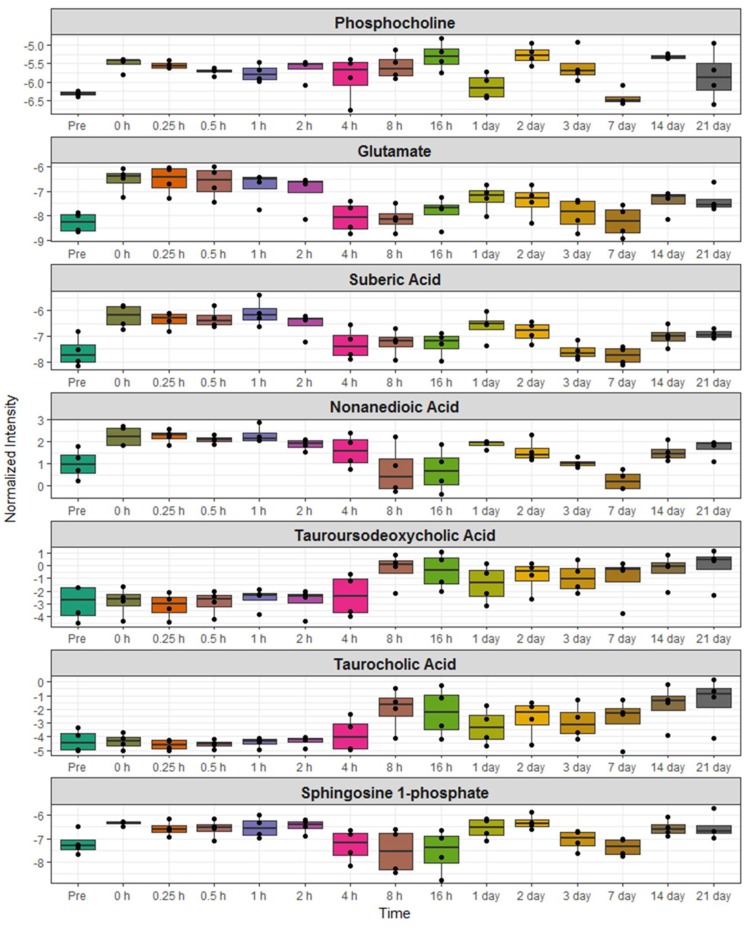
Time dependent transient changes in serum metabolite profiles that are unique to *im* administration of BIO 300 in NHPs.

**Figure 5:**
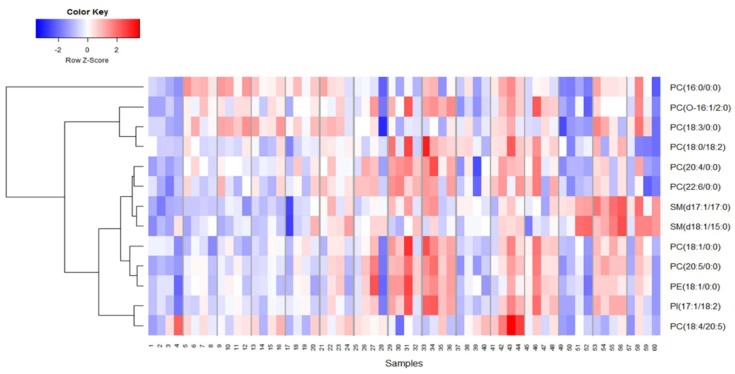
Longitudinal changes in lipid profiles unique to *im* administration of BIO 300 in NHPs indicate that most changes are transient.

**Figure 6:**
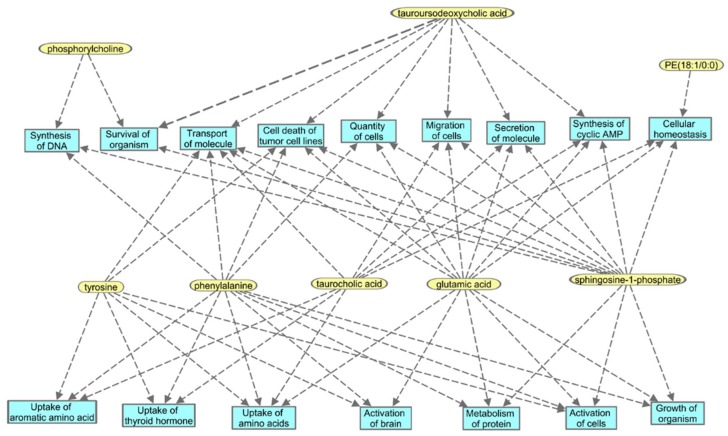
Functional pathway analyses for transient changes in metabolite expression identified changes in glycerophospholipid and bile acid metabolism in NHPs that received *im* BIO 300.

Next, we investigated unique metabolic changes in response to the route of BIO 300 administration. First, we performed ANOVA analysis to delineate metabolites and lipids that showed statistically significant changes following *im* administration at post-administration time points as compared to pre-dosing (Figure 4 and Figure 5) and confirmed their identity using tandem mass spectrometry. As expected, for statistically significant changes, we observed modest fold changes in metabolites including amino acids glutamate, suberic acid, taurodeoxycholic acid and nonedioic acid. Remarkably, the relative abundance of these metabolites showed a modest increase at early time points (up to 4 h) followed by an oscillatory pattern showing a relative decrease followed by a relative increase in serum levels of these metabolites. The predominant class of lipids that showed a modest change included glycerophospholipids and sphingomyelin (Figure 5). Pathway analysis of metabolites with altered expression patterns showed changes in phospholipid, bile acid and amino acid metabolism (Figure 6).

**Figure 7:**
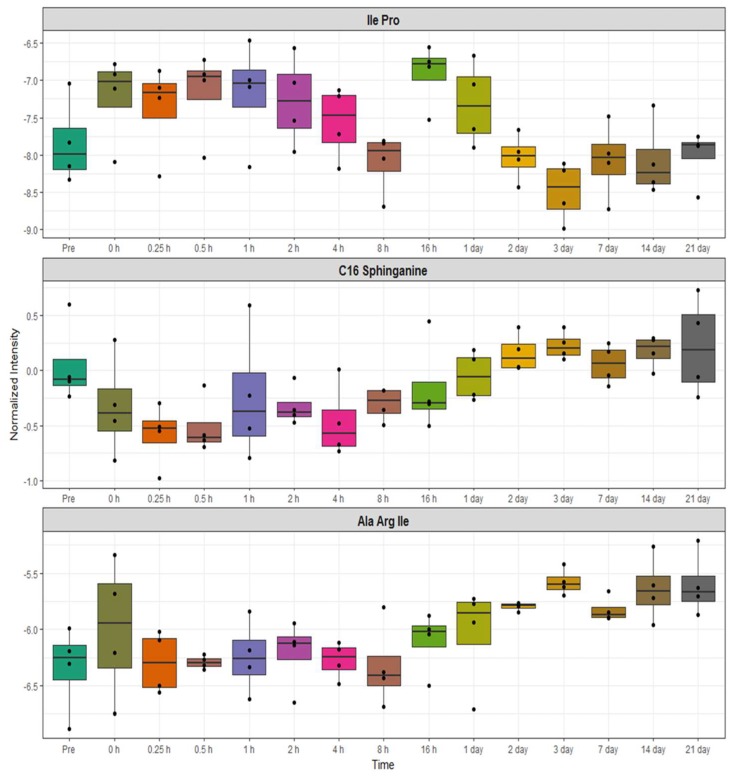
Box and whisker plot representation of oscillatory changes in serum metabolite profiles up to 21 days after oral administration of BIO 300.

**Figure 8:**
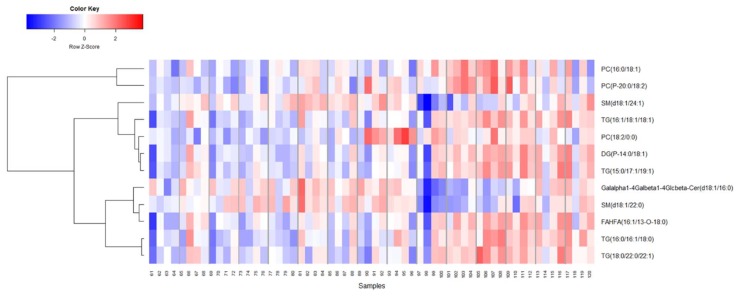
Hierarchical clustering shows longitudinal changes in sphingomyelin and glycerophosphocholine classes of lipids in NHPs that received BIO 300 administered *po*.

**Figure 9:**
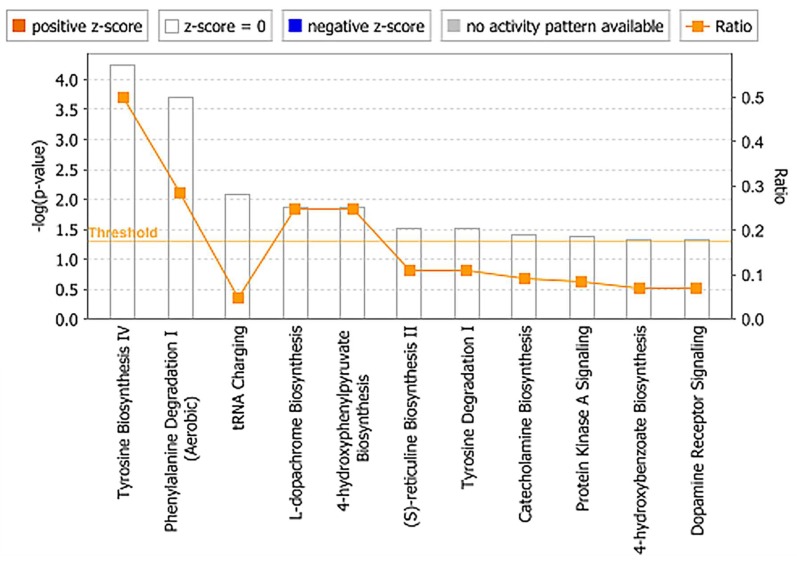
Ingenuity functional pathway analysis shows changes in tyrosine biosynthesis and phenylalanine degradation pathways after *po* administration of BIO 300 in NHPs.

Secondly, we performed similar analyses to determine changes in serum metabolites and lipid profiles in NHPs receiving BIO 300 through the *po* route of administration. This cohort showed minimal metabolic changes post-administration overtime (Figure 7 and Figure 8). We observed modest changes in di- and tripeptide and C16 sphingosine levels as well as transient changes in serum levels of triglycerides, glycerophospholipids and sphingomyelins, predominantly between 3–7 days after drug treatment while the levels reverted to pre-treatment levels by day 7–14 post administration for most metabolites. Pathway analysis of metabolites with altered patterns of expression indicated upregulation of tyrosine biosynthesis and phenylalanine degradation as the two top BIO 300 targeted pathways (Figure 9).

## 3. Discussion

Individuals exposed to high doses of acute radiation face life threatening injury. This is especially relevant to the members of our armed services and first responders who are most at risk of exposure during a nuclear attack or disaster [26,27,28]. To date, the FDA has approved three drugs (Neupogen^®^, Leukine^®^, and Neulasta^®^) to treat H-ARS which are only effective after individuals have been exposed to radiation. Unfortunately, no prophylactic/preventative agents are FDA approved to prevent either ARS or DEARE [4,5,6,7,8]. 

We are developing a radioprotective agent, genistein, which is able to prevent lethality associated with H-ARS, if administered before radiation exposure and also mitigates delayed pulmonary effects of lethal radiation exposure (DEARE-lung), if administered post radiation exposure. Genistein is a challenging molecule to manufacture into a drug product; it is nearly insoluble in most excipient systems and has very poor *po* bioavailability. To overcome these limitations, we developed a patent-protected *po* suspension formulation that contains synthetic genistein wet-nanomilled into nanoparticles. A dose escalating pharmacokinetic study in canines demonstrated greatly improved oral bioavailability of genistein delivered as nanoparticles in BIO 300 compared to non-nanomilled genistein (unpublished). In this study an approximate 3–7 fold increase in total drug exposure (AUC) and a 6–10 fold increase in Cmax were observed in animals dosed with BIO 300 compared to non-nanomilled genistein. A direct comparison using the *im* route of administration is not feasible. The genistein particle size in a non-nanomilled formulation prohibits administration by this route.

This genistein nanosuspension (BIO 300) is in an advanced stage of development for prevention of H-ARS and has an open Investigational New Drug application (IND–74460). In addition, BIO 300 is currently being evaluated for safety, pharmacokinetics, and efficacy in a Phase 1b/2a clinical trial in non-small cell lung cancer (NSCLC) patients (NCT02567799). The focus of this study is to prevent or mitigate the delayed effects of acute radiation exposure, specifically, the life-threatening side-effects (pneumonitis and pulmonary fibrosis) resulting from radiation exposure to the normal lung tissue.

A variety of systemic administration routes can be used to deliver therapeutic drugs. Effort must be made to select the route of administration that delivers the drug to the site of action in a timeframe that aligns with the clinical indication. Thus, uptake or absorption into the bloodstream is the critical first step for the drugs response. A parenteral route of drug administration (e.g., *im* administration) is typically used to avoid the gastrointestinal tract for drugs that may not be stable in the gastrointestinal tract or drugs that are unable to penetrate the gastrointestinal membrane. Parenteral administration often results in the drug reaching the bloodstream faster than orally administered drugs. However, drugs that are administered by this route may be more gradually released into the bloodstream compared to orally delivered drugs due to capillary membrane structure and paracellular penetration at the site of injection. 

We found a significant increase in the Cmax (~7 fold increase) and AUC (~16 fold increase) of the active form of genistein (aglycone) following a single intramuscular injection of BIO 300 compared to a single oral administration of the drug at twice the *im* dose (50 mg/mg im or 100 mg/kg, *po*). The doses of BIO 300 evaluated in this study were allometrically scaled from rodent efficacy studies demonstrating BIO 300-mediated radioprotection or radiomitigator properties [15,16]. Moreover, the BIO 300 doses being evaluated in the ongoing NSCLC clinical trial (NCT02567799) were also determined using allometric scaling (500, 1000, 1500 mg). Studies in large animals (e.g., NHP) need to be completed to unequivocally demonstrate the radioprotection/radiomitigator effects of BIO 300 administered at these doses. Importantly, the blood levels genistein aglycone obtained in this study by either route of administration are sufficient to activate the drugs’ biological target (ERβ), which has an IC50 of 8.4 nM [18]. ERβ is found throughout the body of both males and females, making it an ideal candidate target for medical countermeasures. 

In vitro studies have found that in mouse, canine and human cells the majority of genistein is glucuronidated, with up to 88% glucuronidated in human cells (unpublished). In this unpublished study, glucuronidated genistein was found in two forms, 7-*O*-glucuronide (~95%) and 4-*O*-glucuronide (~5%). The assay used in the current NHP study measures genistein aglycone in a serum sample prior to and post treatment with β-glucuronidase. Sample chromatograms of the genistein metabolite (genistein 7-*O*-glucuronide) and of the genistein measured in NHP serum are provided for reference as Figures S6 and S7, respectively). The measurement of total genistein allows for a determination of the fraction of drug that is in the aglycone form which is the active form of the drug. Importantly, genistein has been shown to be converted to its inactive glucuronidated form in the gut, resulting in lower levels of the active genistein aglycone form in the circulation [29]. This was in fact what was observed in our NHP study, where significantly higher levels of genistein aglycone were observed in the circulation following *im* administration. Moreover, the absence of similar BIO 300 absorption rate (Tmax) across all animals and extent of absorption (Cmax) can be attributed to the inherent variability of parenteral administration which results in variations of PK measures, such as AUC, within a single individual and between individuals of the same species. Others have also noted the variability of subcutaneous (sc) administration (e.g., administration of cefovecin to NHPs demonstrated intra-individual coefficient of variations in PK parameters which were significantly higher than those with intravenous administration) [30,31]. Therefore, the cefovecin comparison described above is more descriptive of the intra-individual differences observed for BIO 300. Depending on BIO 300’s use case as a radiation countermeasure and the desired PK (e.g., Cmax, AUC), the data described here suggest broad therapeutic applicability for BIO 300. For example, a single *im* administration could be used to obtain and sustain serum levels of genistein aglycone capable of activating the drug’s biological target for an extended period of time while oral dosing could be used to achieve a rapid increase in blood levels for an immediate (short duration) activation of the target.

One of the outstanding questions for drug safety studies pertains to impact on overall metabolism that may either interfere with the intended use of the product and/or cause molecular changes that may be deleterious to the overall physiology of humans. Hence, an added goal of our study was to conduct a comprehensive investigation of metabolomic and lipidomic changes to delineate specific changes after administration of BIO 300 by the two routes of administration. We used NHPs as a model system to compare longitudinal changes in molecular metabolomic and lipidomic profiles in these animals following either *im* or *po* administration of BIO 300. Our results show that the administration of the drug results in a modest change in the metabolic/lipidomic profiles over time. More importantly, the observed changes are most apparent between days 3–7 post-BIO 300 administration and subsequently revert to normal levels during the remainder of the experimental period (monitored up to 21 days). Pragmatically, 31 days is considered a wash out period for this drug, which coincides with the metabolic homeostasis observed by day 21. Modest increases were observed in serum levels of amino acids, glycerophospholipids and sphingomyelins. This could be attributed in part to an increased bioavailability of these metabolites as a possible radioprotective mechanism since lipids and proteins are susceptible to radiation induced cell damage [32,33,34]. In addition, elevated levels of docohexanoic acid (shown to have strong anti-oxidant activity) and cholic acid (which is reported to inhibit lipid peroxidation) could provide overall metabolic benefit to animals receiving the drug [35]. We also found a significant overlap of the metabolic changes after the two routes of administration. This suggests that these transient changes in overall metabolism are independent of the route of BIO 300 administration. Overall, treatment with BIO 300 resulted in less than 10% change in serum levels of the assayed metabolites and lipids; although the apparent inter-group fold change for some of the lipids was in the range of 1.6 fold, there was a huge intra-group variance at any time point. Interestingly, we found fewer changes following *po* administration as compared to *im* administration of BIO 300. Taken together these investigations enabled us to gain insights into biochemical perturbations in response to BIO 300 administration, which could be attributed in part to the radioprotective effects observed. Additional follow on studies will be aimed at further elucidating the role of the observed metabolite and lipid changes to determine their impact on medical countermeasure efficacy. The fact that minimal perturbations were observed with the two routes of administration tested supports the safety of the drug.

Recently, a metabolomic study was conducted to identify tissue-derived biomarkers that correlate with radiation-induced lung injury and BIO 300 efficacy for mitigating tissue damage. High-throughput targeted metabolomics of lung tissue samples obtained from male C57L/J mice exposed to 12.5 Gy whole thorax lung irradiation and treated with BIO 300 (400 mg/kg daily for either 2 or 6 weeks starting 24 h post-irradiation) were analyzed at 180 day post-irradiation. A panel of lung metabolites (amino acids such as Asp, Glu, Glyn, Asn, Ser, Thr, Trp, Tyr, ProVal, and lipids: diacyl glycerophosphatidylcholine (PCa), ether glycerophosphatidylcholine (PCe), and sphingomyelin) that are responsive to radiation and able to distinguish an efficacious treatment schedule of BIO 300 from a non-efficacious treatment schedule in terms of 180 day survival were identified [36].

Nonclinical studies have evaluated daily dosing durations of BIO 300 for up to 10 weeks with no significant adverse effects or toxicity concerns [15]. The drug is being evaluated in an ongoing clinical trial in non-small cell lung cancer patients (NCT02567799). In this study, BIO 300, at one of three daily doses (500, 100, 1500 mg), is administered for up to 8 weeks during the entire course of concurrent chemoradiotherapy. These studies will be used to support the dose and dosing duration for potential medical countermeasure applications. Toward that goal, BIO 300 is being developed as an effective radioprotectant for ARS and a radioprotectant/mitigator for DEARE. The development of a single agent to prevent and/or mitigate multiple organ syndromes following radiation exposure would be extremely advantageous to first responders and military personnel as it would eliminate the potential for drug–drug interactions as well as minimize the risk of adverse reactions and contraindicated maladies. 

## 4. Materials and Methods

### 4.1. Animals and Animal Care

Four naïve rhesus macaques (*Macaca mulatta*, Chinese sub strain, two males and two females) 3–7 years of age, weighing 5 to 7 kg, were obtained from National Institutes of Health Animal Center (NIHAC, Poolesville, MD, USA) and maintained in a facility accredited by the Association for Assessment and Accreditation of Laboratory Animal Care (AAALAC)-International. Animals were quarantined for six weeks prior to initiation of the experiment. Animal housing, health monitoring, care, and enrichment during the experimental period have been described earlier [37]. Animals were fed a primate diet (Teklad T.2050 diet; Harlan^®^ Laboratories Inc., Madison, WI, USA) twice daily with at least six hours between feedings (animals were fed four biscuits each at 7:00 AM and 2:00 PM) and received drinking water *ad libitum*. All procedures involving animals were approved (Protocol # P2017-02-005 approved on 23 February 2017) by the Armed Forces Radiobiology Research Institute Institutional Animal Care and Use Committee (IACUC) and Department of Defense Animal Care and Use Review Office (ACURO). This study was carried out in strict accordance with the recommendations in the Guide for the Care and Use of Laboratory Animals of the National Institutes of Health [38].

### 4.2. Drug Preparation and Administration

Two BIO 300 formulations were used in this study. BIO 300 Injectable Suspension (323 mg/mL genistein, 5% povidone K17 (*w*/*w*), 0.2% polysorbate 80 (*w*/*w*) in 50 mM phosphate buffered saline (61 mM sodium chloride)) was used for *im* dosing and BIO 300 Oral Suspension (325 mg/mL genistein, 5% povidone K25 (*w*/*w*), 0.2% polysorbate 80 (*w*/*w*), 0.18% methylparaben (*w*/*w*), and 0.02% propylparaben (*w*/*w*)) was used for *po* dosing. These two formulations have an identical active pharmaceutical ingredient, synthetic genistein nanoparticles, but differ according to their excipients, which have been optimized for either oral or parenteral use. Four NHPs were administered a single dose of BIO 300 Injectable Suspension (50 mg/kg) *im* using a 23 G needle length of 5/8” attached to a 1 mL syringe. The site for injection was prepared as a surgical site before the injection: hair was clipped using #40 surgical blade and the site was scrubbed at least three times using 4% chlorhexidine and 70% alcohol. The same NHPs used for the PK *im* study were used for the PK *po* study after their 31 day wash-out period. BIO 300 Oral Suspension (100 mg/kg) was administered *po* as a single dose via nasogastric (NG) tube. NG tube placement was confirmed via digital X-ray prior to drug administration. 

### 4.3. Serum Sample Collection

Blood was collected by venipuncture from the saphenous vein of the lower leg after the site was cleaned using a 70% isopropyl alcohol wipe and dried with sterile gauze. All animals were restrained using the pole-and-collar method and placed in a chair for blood collection. On the day of drug administration, animals were bled repeatedly at 0, 0.25, 0.5, 1, 2, 4, 8, and 16 h post-drug administration. On days when animals were only bled once, the blood draw was conducted between 08:00 AM and 10:00 AM, 1–3 h after animals were fed. The relation from the time of feeding to any specific bleeding was consistent for all animals. The desired volume of blood was collected with a 3 mL disposable luer-lock syringe with 25-gauge needle. For serum collection, the blood sample was transferred to Capiject serum separator tubes (3T-MG; Terumo Medical Corp, Elkton, MD, USA), and allowed to clot for 30 min, then centrifuged (10 min, 400× *g*). Serum samples were stored at −70 °C until blood biochemistry analysis or shipped on dry ice to Bioanalytical Systems, Inc. (BASi, West Lafayette, IN, USA) for PK analysis, or to the Georgetown University Medical Center (Washington, DC, USA) for the metabolomic study. Blood samples for CBC were collected in EDTA (ethylenediaminetetraacetic acid) blood collection tubes (Sarstedt Inc., Newton, NC, USA) and mixed in a rotary shaker. 

### 4.4. Pharmacokinetic Analysis

The PK samples from both studies were analyzed by Bioanalytical Systems, Inc. Genistein aglycone was extracted from NHP serum by liquid/liquid extraction with ethyl acetate (EtOAc). Prior to extraction, genistein-d4, which is an isotope-labelled drug, was added as an internal standard. The organic layer was collected, transferred to a new plate, and evaporated to dryness. The residue was reconstituted with a water/acetonitrile/formic acid mixture and injected into a liquid chromatography-tandem mass spectrometry system (LC-MS/MS) using a Betasil C18 column with a water/acetonitrile/formic acid mobile phases. 

Total genistein (glucuronidated genistein and genistein aglycone) was extracted from NHP serum samples after incubation at 38 °C with β-glucuronidase for one hour to generate genistein aglycone, followed with the same extraction and LC-MS/MS method. As control, blank NHP serum sample was obtained from BioChemed (Winchester, VA, USA) and stored at −80 ± 20 °C and thawed before use at room temperature. Data acquisition was performed using Analyst software (SCIEX, Redwood City, CA, USA). Regression and calculation of results and statistics were performed using Watson^®^ LIMS version 7.3.0.01 (Thermo Fisher Scientific, Waltham, MA, USA).

### 4.5. Serum Metabolomics Using UPLC QTOF Analysis 

Serum was prepared for metabolomic analysis as described previously [39,40]. Briefly, metabolite extraction was performed by adding 75 µL of 40% isopropanol (IPA) + 25% methanol + 35% water containing internal standards to 25 µL of NHP serum. Samples were vortexed and incubated on ice for 20 min followed by the addition of 100 µL of acetonitrile (ACN) to precipitate proteins. Samples were incubated at −20 °C for 15 min and centrifuged at 13,000 rpm at 4 °C for 20 min. Supernatant was transferred to fresh vials for UPLC-ESI-Q-TOF-MS analysis.

For metabolomic analysis, each sample (2 μL) was injected onto a reverse-phase 50 × 2.1 mm Acquity 1.7 μm BEH C18 column at 60 °C column temperature (Waters Corp, Milford, MA, USA) using an Acquity UPLC system (Waters) with a gradient mobile phase consisting of 100% water containing 0.1% formic acid (Solvent A) and 100% ACN containing 0.1% formic acid (Solvent B) and 90% IPA + 10% ACN containing 0.1% formic acid (Solvent C), and resolved for 13 min at a flow rate of 0.5 mL/min. The gradient started with 98% A and 2% B for 0.5 min with a ramp of curve 6. At 4 min, the gradient reached 40% A and 60% B. At 8 min, the gradient shifted to 2% A and 98% B for one min. From 9.5 to 11 min, the gradient was 98% C and 2% B. At 11.50 min, it shifted to 50% A and 50% B. At 12 min, it reached initial conditions. 

The column eluent was introduced directly into the mass spectrometer by electro-spray. Mass spectrometry was performed on a Q-TOF MS (Xevo G2 QTOF MS, Waters Corporation, Milford, MA, USA), operating in either negative-ion (ESI) or positive-ion (ESI) electro-spray ionization mode with a capillary voltage of 3 kV for positive mode and 1.5 kV for negative mode and a sampling cone voltage of 30 V in both negative and positive modes. The extraction cone was 3.0. The desolvation gas flow was set to 1000 L/h and the temperature was set to 500 °C. The cone gas flow was 25 L/h, and the source temperature was 120 °C. Accurate mass was maintained by introduction of LockSpray interface of Leucine Enkephalin (556.2771 [M+H]+ or 554.2615 [M-H]−) at a concentration of 2 ng/μL in 50% aqueous ACN and a rate of 5 μL/min. Data were acquired in centroid mode from 50 to 1200 *m*/*z* in MS scanning. Pooled QC (quality control samples) were run throughout the batch to monitor data reproducibility. The identification of a sub-set of metabolites was confirmed using fragmentation pattern matching using tandem mass spectrometry (Table S1).

### 4.6. Analysis for CBC

Total white blood cells (WBC), erythrocytes (red blood cells (RBC)), platelets, neutrophils, lymphocytes, monocytes, reticulocytes, basophils, hemoglobin (HGB), and hematocrit (HCT) were counted using an Advia 120-cell counter (Bayer Corporation, Tarrytown, NY, USA) [37]. 

### 4.7. Data Processing and Statistical Analysis

Centroided and integrated mass spectrometry data from the UPLC-TOFMS were preprocessed using XCMS software (Scripps Research Institute, La Jolla, CA, USA) to generate a data matrix containing ion intensities, mass to charge (*m*/*z*) and retention time values. The data were normalized to the intensities of internal standards. Multivariate statistics were performed using R scripts developed in-house [41]. ANOVA comparison was used to identify significantly BIO 300 targeted metabolites (based on *m*/*z* values) between comparative groups and further corrected using the Benjamini-Hochberg (FDR) multiple testing correction method. The identity of significant changes in metabolite expression was confirmed using tandem mass spectrometry (Table S1). Additionally, the identity of lipids were confirmed using SIMPLIPID software V6.01 (Premier Biosoft, Palo Alta, CA, USA), by fragmentation pattern matching. Pathway analysis was performed using the Ingenuity Pathway Analysis (IPA) software (Qiagen, Hilden, Germany). 

For CBC and blood biochemistry data, mean values with standard errors (SE, when applicable) are reported. Paired sample *t*-tests were used to detect if there were significant differences between pre- and post-BIO 300 administration time points. All statistical tests were two-sided, with a 5% significance level. Statistical software SPSS version 22 (IBM, Armonk, NY, USA) and GraphPad Prism 5 (GraphPad Software, Inc., La Jolla, CA, USA) were used for analyses.

## Figures and Tables

**Table 1:** PK *im* study sample analysis of free and total genistein.

***im* Free Genistein (Aglycone) (50 mg/kg)**					
**NHP**	**Tmax (hr)**	**Cmax (ng/mL)**	**T_1/2_ (h)**	**AUC_0–48_ (ng.h/mL)**	**AUC_0–∞_ (ng.h/mL)**
NHP 1	2	3550	5.47	24,159	24,234
NHP 2	1	1980	15.26	44,783	50,024
NHP 3	2	1410	18.15	28,194	33,667
NHP 4	2	1580	20.17	30,202	36,953
Average	1.75	2130	14.76	31,834	36,219
SD	0.50	976	6.51	8991	10,666
Med	2.00	1780	16.70	29,188	35,310
Max	2.00	3550	20.17	44,783	50,024
Min	1.00	1410	5.47	24,159	24,234
%CV	28.57	45.84	44.11	28.24	29.45
***im* Total Genistein (50 mg/kg)**					
**NHP**	**Tmax (hr)**	**Cmax (ng/mL)**	**T_1/2_ (h)**	**AUC_0–48_ (ng.h/mL)**	**AUC_0–∞_ (ng.h/mL)**
NHP 1	4	5760	6.05	49,046	49,358
NHP 2	8	3570	15.33	83,671	91,588
NHP 3	2	2950	18.91	53,329	64,788
NHP 4	2	2360	19.92	49,330	60,051
Average	4.00	3660	15.05	58,844	66,446
SD	2.83	1485	6.31	16,666	17,961
Med	3.00	3260	17.12	51,330	62,419
Max	8.00	5760	19.92	83,671	91,588
Min	2.00	2360	6.05	49,046	49,358
%CV	70.71	40.56	41.94	28.32	27.03

**Table 2:** PK *po* study sample analysis of free and total genistein.

***po* Free Genistein (Aglycone) (100 mg/kg)**					
**NHP**	**Tmax (hr)**	**Cmax (ng/mL)**	**T_1/2_ (h)**	**AUC_0–48_ (ng.h/mL)**	**AUC_0–∞_ (ng.h/mL)**
NHP 1	4	398	2.57	3041	3048
NHP 2	2	273	2.08	1195	1201
NHP 3	2	162	4.22	1304	1316
NHP 4	2	411	1.84	2127	2133
Average	2.50	311	2.68	1917	1924
SD	1.00	117	1.07	857	856
Med	2.00	336	2.32	1715	1724
Max	4.00	411	4.22	3041	3048
Min	2.00	162	1.84	1195	1201
%CV	40.00	37.69	40.01	44.73	44.50
***po* total genistein (100 mg/kg)**					
**NHP**	**Tmax (hr)**	**Cmax (ng/mL)**	**T_1/2_ (h)**	**AUC_0–48_ (ng.h/mL)**	**AUC_0–∞_ (ng.h/mL)**
NHP 1	4	10,400	2.37	60,788	60,805
NHP 2	2	6330	2.88	38,775	38,912
NHP 3	2	5150	4.09	54,492	54,860
NHP 4	2	5560	2.19	29,703	29,789
Average	2.50	6860	2.88	45,939	46,092
SD	1.00	2410	0.86	14,243	14,267
Med	2.00	5945	2.63	46,633	46,886
Max	4.00	10,400	4.09	60,788	60,805
Min	2.00	5150	2.19	29,703	29,789
%CV	40.00	35.13	29.70	31.00	30.95

## Supplementary Materials

Supplementary materials can be found at https://www.mdpi.com/1422-0067/20/5/1231/s1.
