# Supplementary material for: Pharmacokinetic and Metabolomic Studies with BIO 300, a Nanosuspension of Genistein, in a Nonhuman Primate Model
Source: Int J Mol Sci. 2019 Mar 12;20(5):1231. doi: 10.3390/ijms20051231 (PMC6429499; doi:10.3390/ijms20051231)
Supplement: Supplementary file 1 [file ijms-20-01231-s001.zip › supplementary figures.pdf]

International Journal of
Molecular Sciences

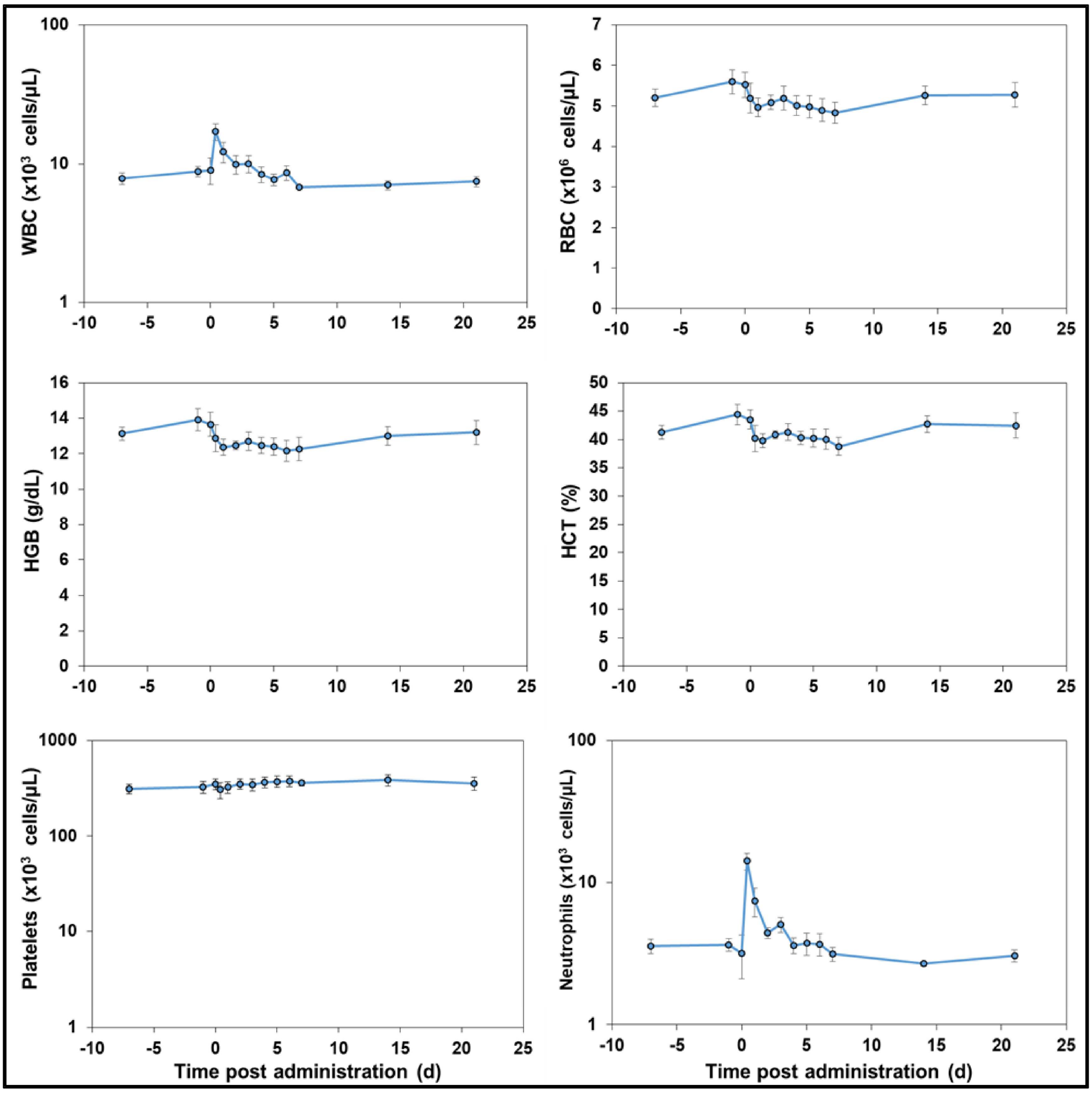

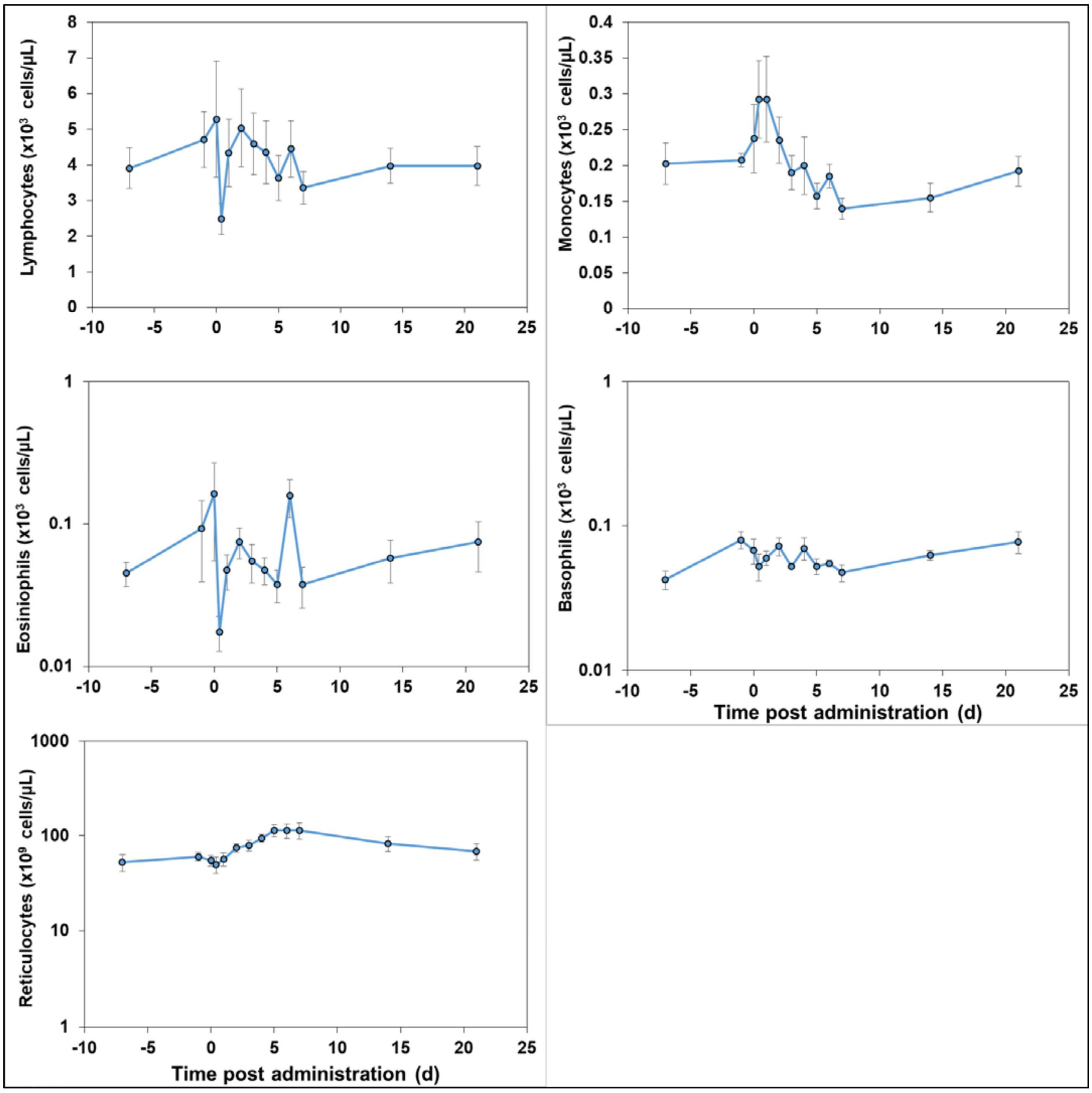
Lymphocytes (x10³ cells/µL)
Monocytes (x10³ cells/µL)
Eosiniophils (x10³ cells/µL)
Basophils (x10³ cells/µL)
Reticulocytes (x10⁹ cells/µL)
Time post administration (d)
Time post administration (d)

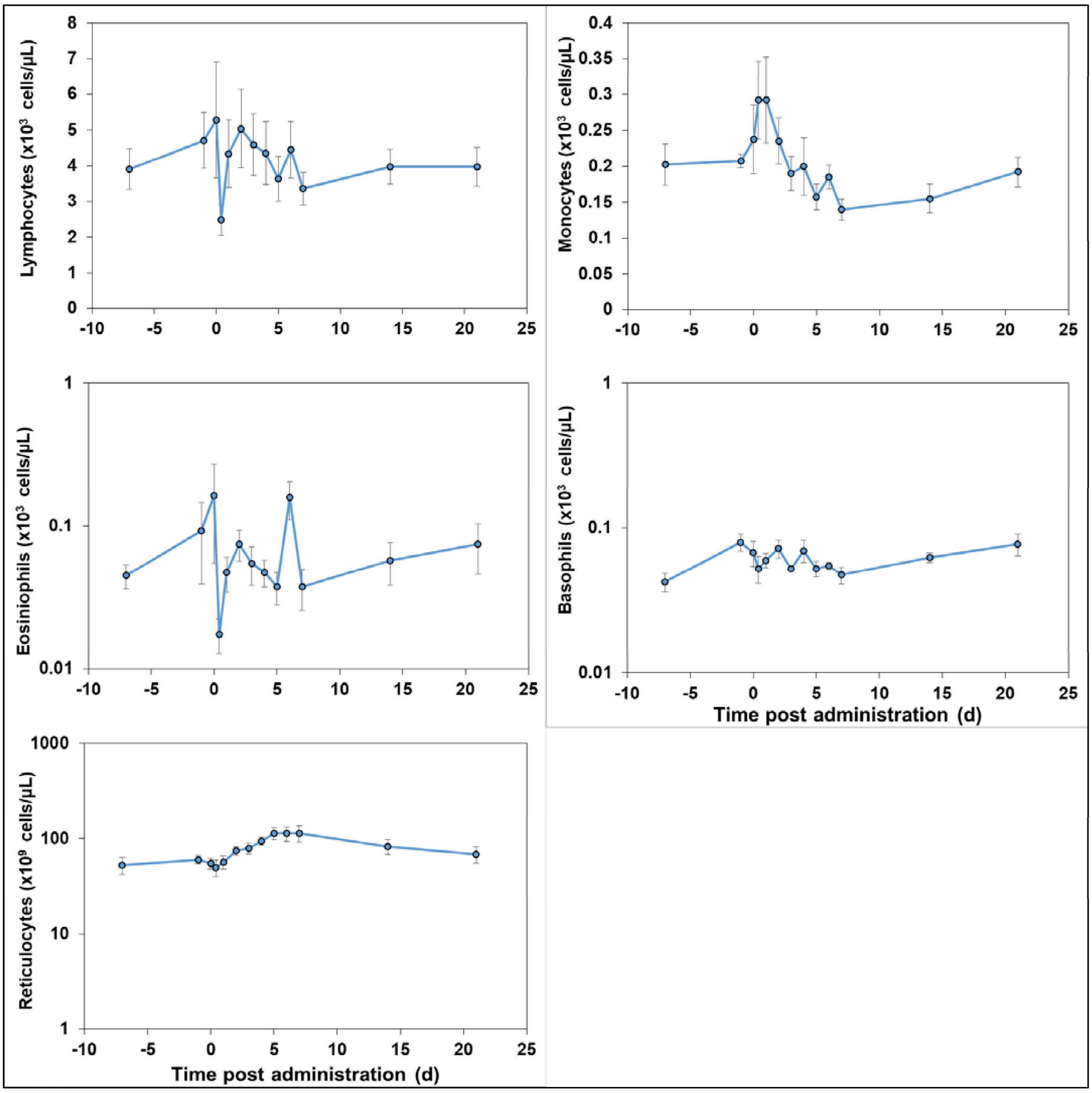

Figure S1. Complete blood count (CBC) following BIO300 *im* administration in NHPs at different time points post drug administration. Four NHPs were administered BIO 300 (50 mg/kg) *im*. Blood was collected at various time points and cells were counted using a Bayer Advia-120 cell counter. The data for each time point is presented as the mean ± standard error of all NHPs.

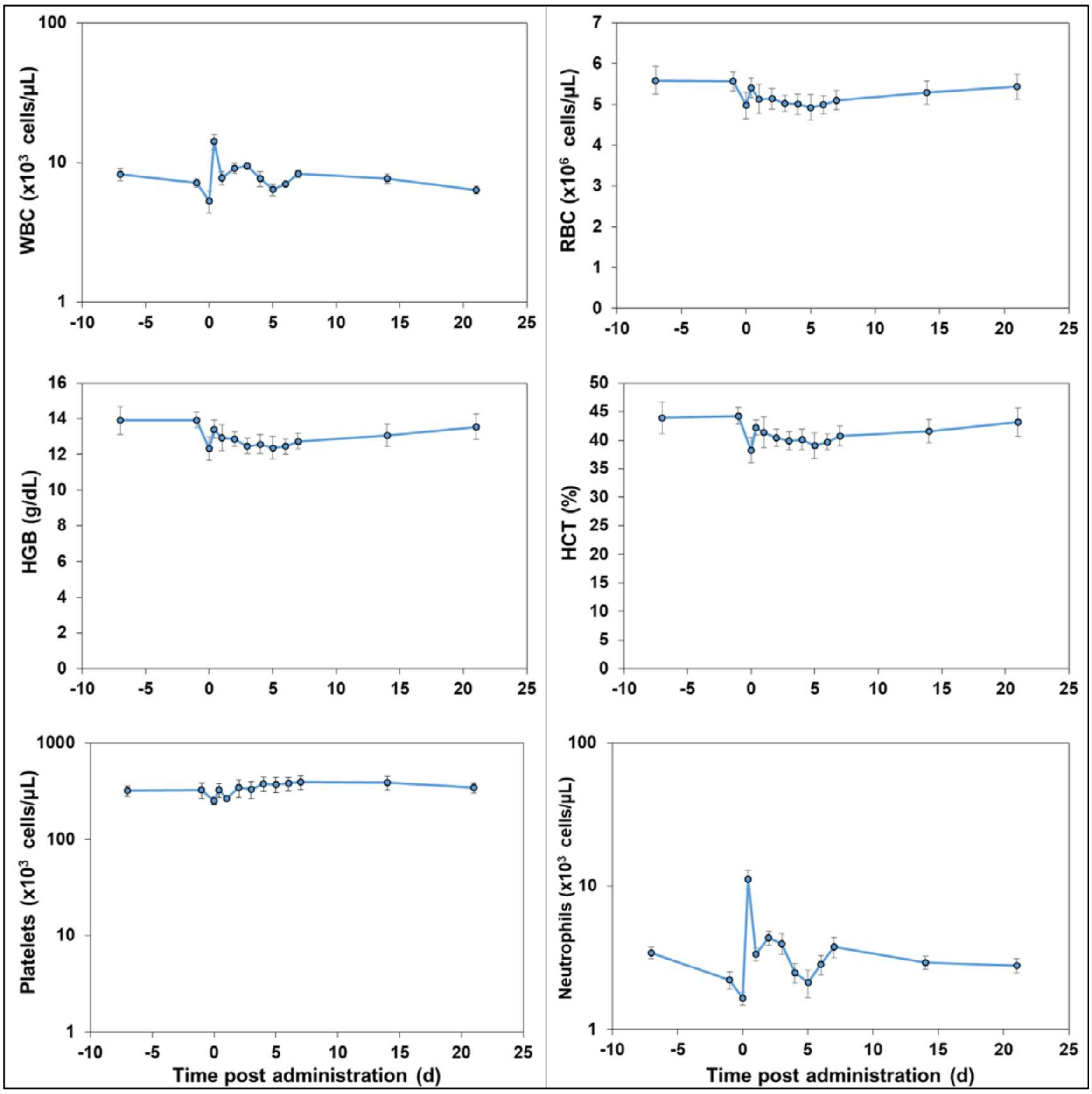
WBC (x10³ cells/μL)
RBC (x10⁶ cells/μL)
HGB (g/dL)
HCT (%)
Platelets (x10³ cells/μL)
Neutrophils (x10³ cells/μL)
Time post administration (d)
Time post administration (d)

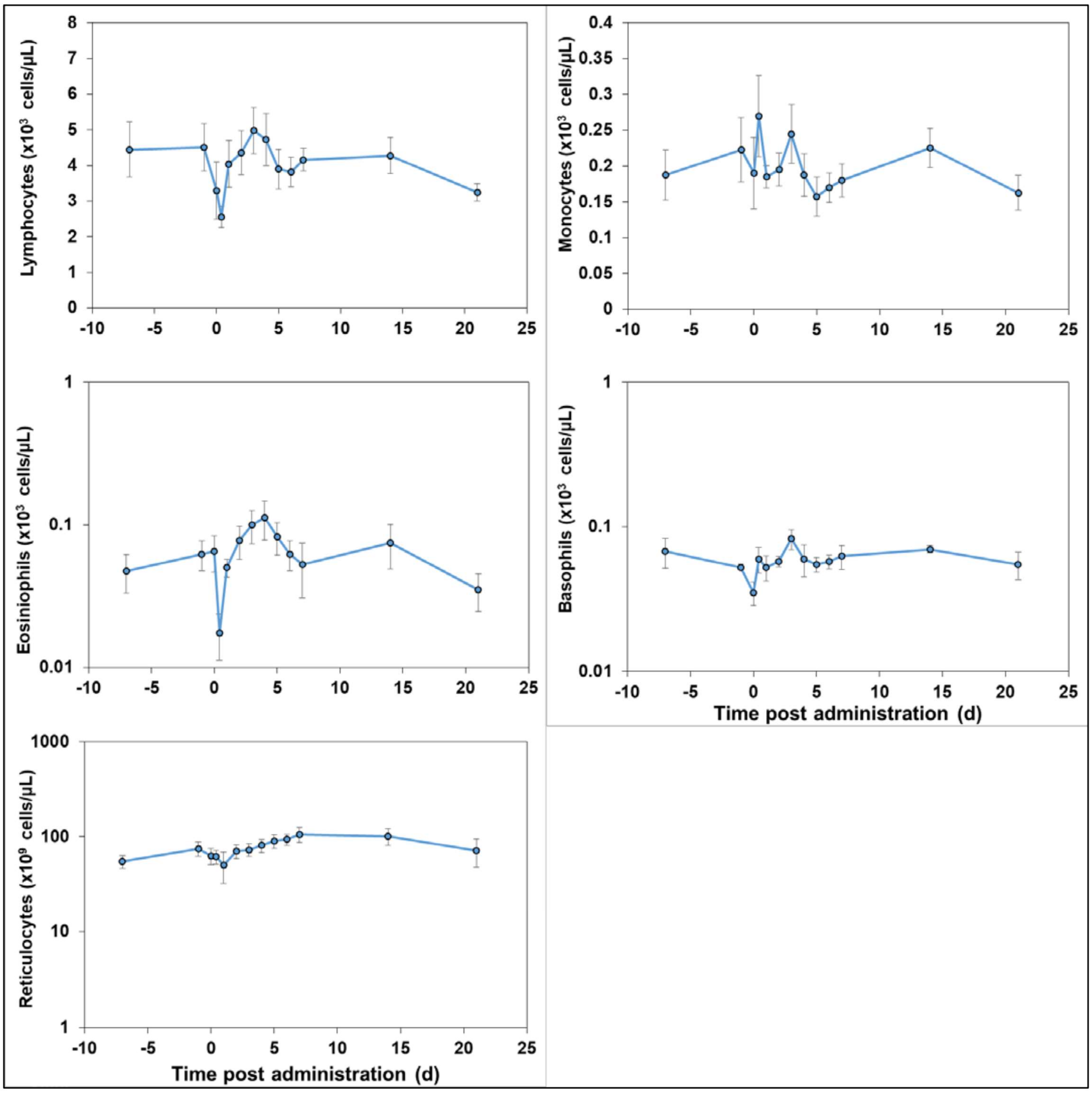

Figure S2. Complete blood count (CBC) following BIO300 *po* administration in NHPs at different time points post drug administration. Four NHPs were administered BIO 300 (100 mg/kg) *po*. Blood was collected at various time points and cells were counted using a Bayer Advia-120 cell counter. The data for each time point is presented as the mean ± standard error of all NHPs.

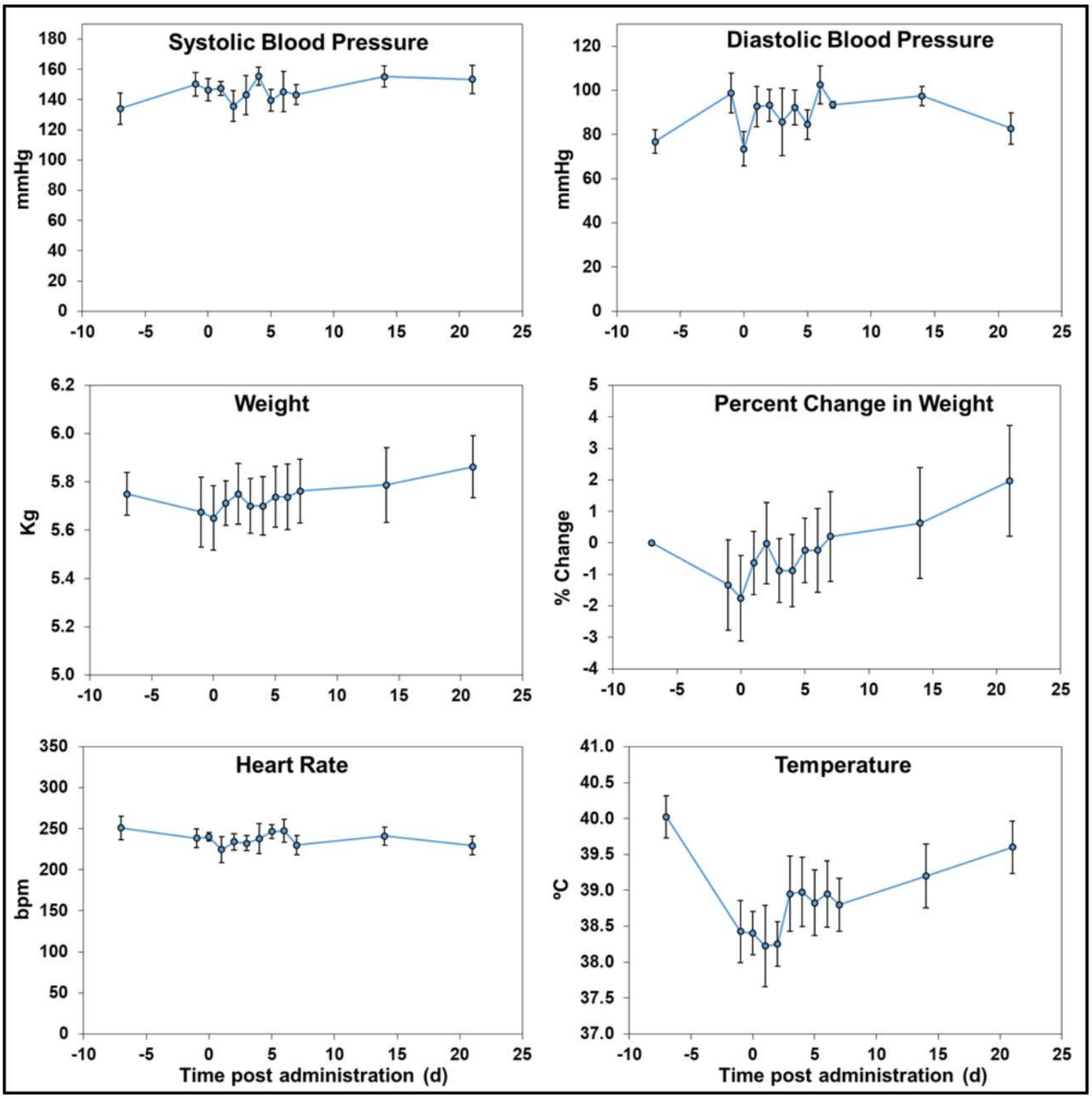

Figure S3. Vital signs collected in NHPs at various time points following BIO 300 *im* administration. Four NHPs were administered BIO 300 (50 mg/kg) *im*. Temperature was taken using a rectal probe SD-7, -4 and SD-1; for the remainder of the time point, temperature was taken using the DAS-7006/7r scanner with implanted chip. Heart rate and blood pressure were taken at various time points using a SurgiVet Advisor vital signs monitor. Weight was taken at various time points using a platform scale. The data for each time point is presented as the mean ± standard error of all NHPs.

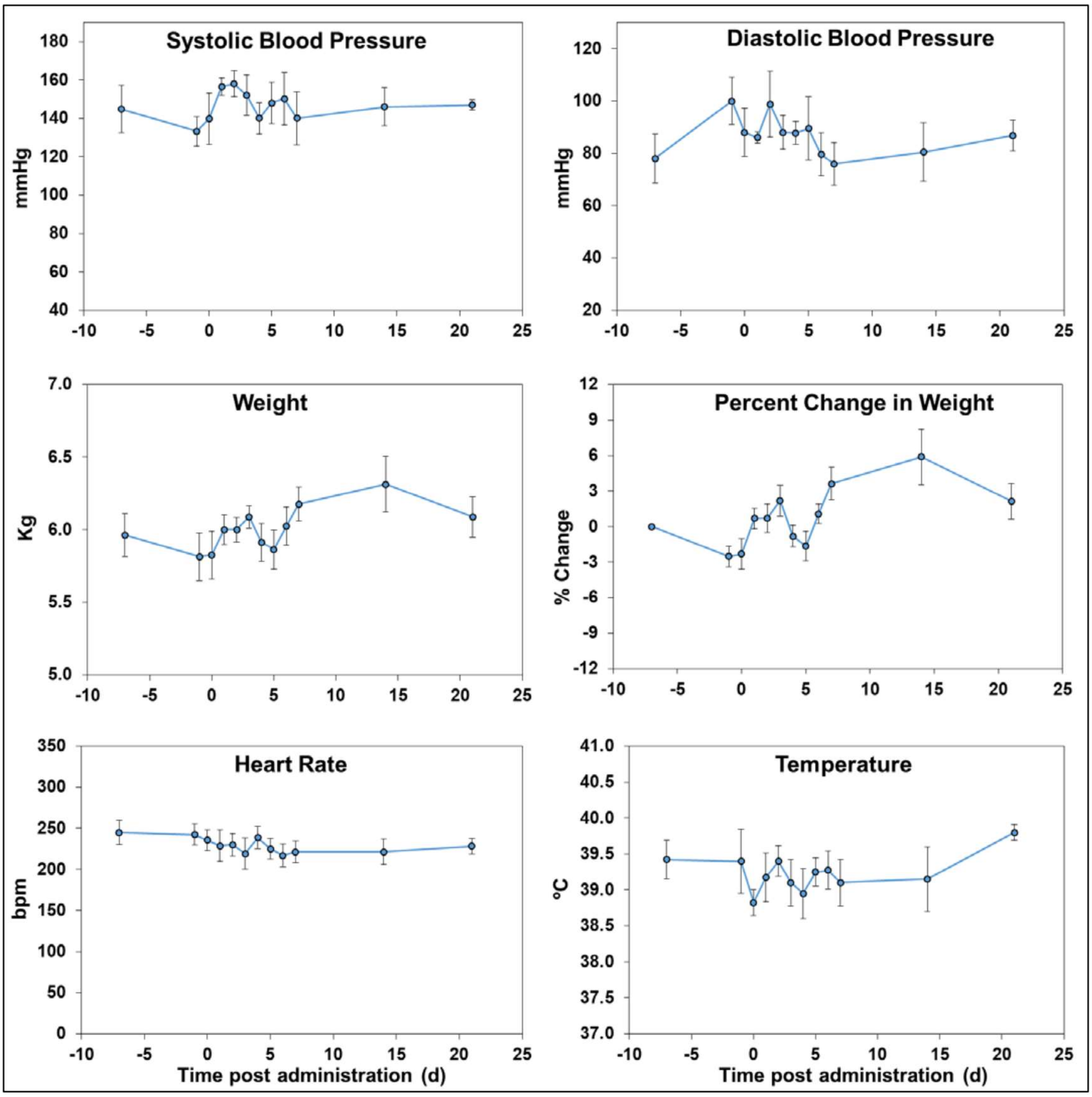

Figure S4. Vital signs collected in NHPs at various time points following BIO 300 *po* administration. Four NHPs were administered BIO 300 (100 mg/kg) *po*. Temperature was taken using a rectal probe SD-7, -4 and SD-1; for the remainder of the time point, temperature was taken using the DAS-7006/7r scanner with implanted chip. Heart rate and blood pressure were taken at various time points using a SurgiVet Advisor vital signs monitor. Weight was taken at various time points using a platform scale. The data for each time point is presented as the mean ± standard error of all NHPs

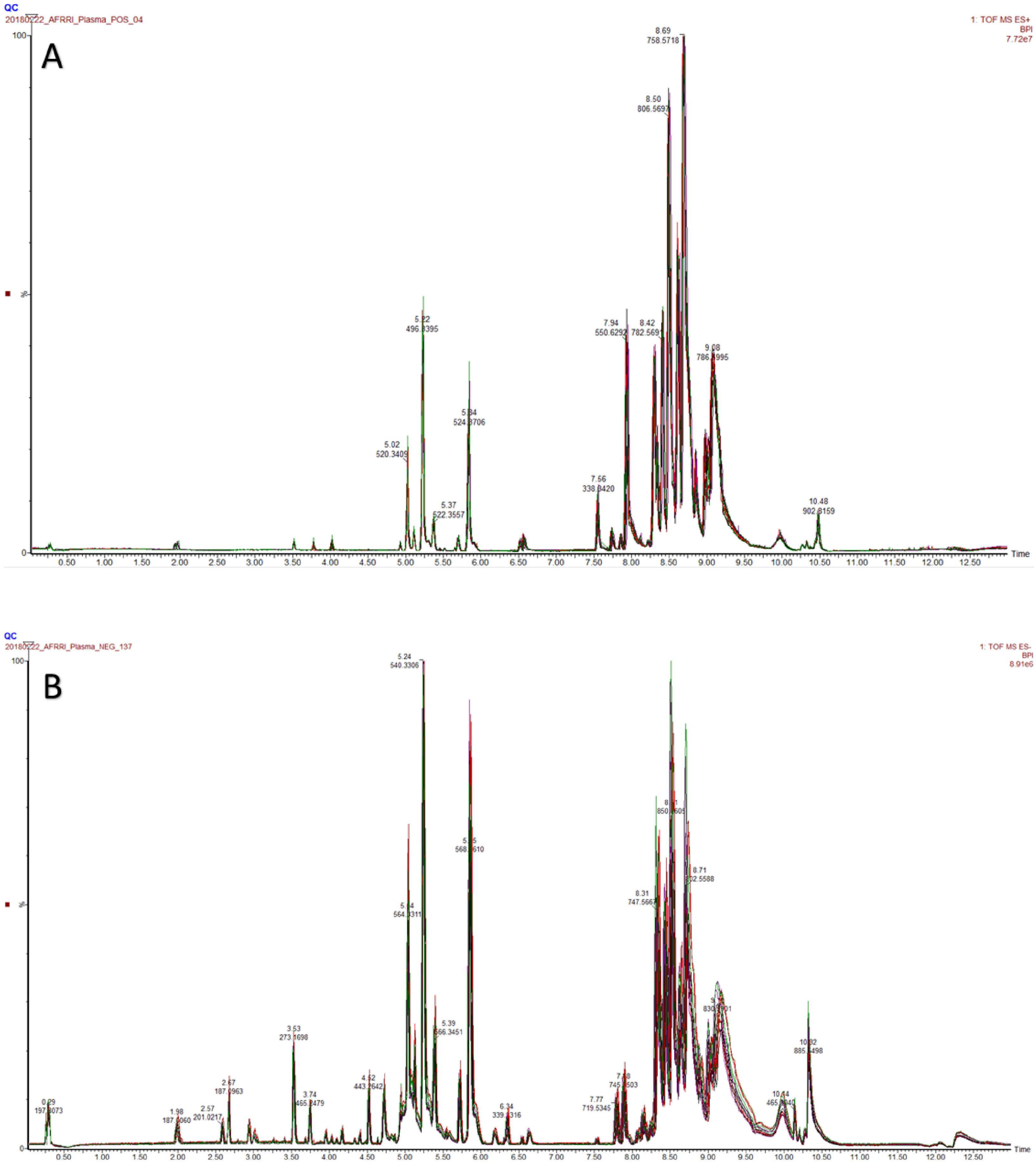

C

Mode	% CV
Positive	22.98%
Negative	11.20%

Figure S5. QC base peak intensity chromatogram overlays in the (**A**) positive mode and (**B**) negative mode, and (**C**) %CV values of the internal standard in QC.

A

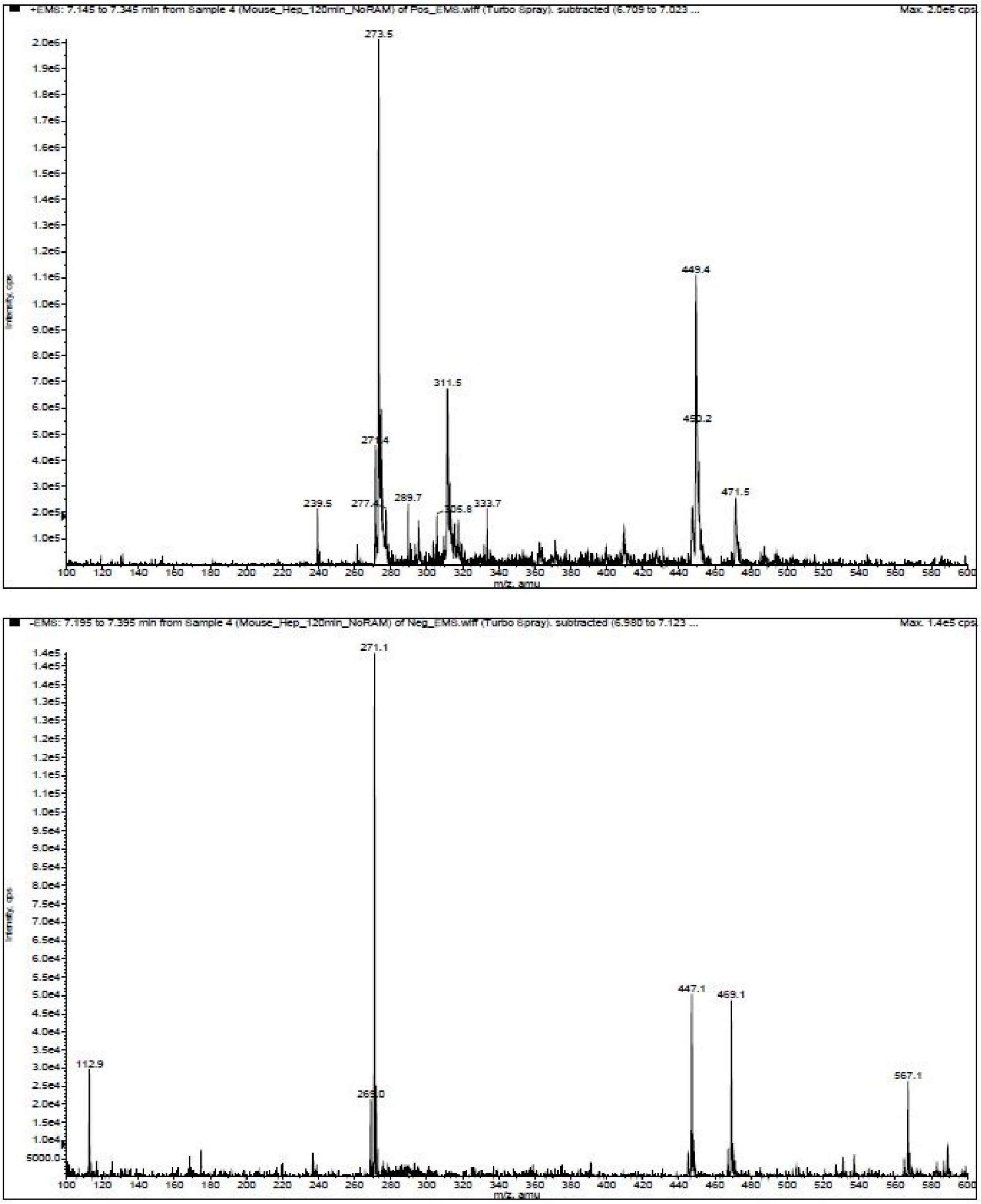
+EMS: 7.145 to 7.345 min from Sample 4 (Mouse_Hep_120min_NoRAM) of Pos_EMS.wiff (Turbo Spray), subtracted (6.709 to 7.023 ...
Max. 2.0e6 cps.
273.5
449.4
311.5
450.2
271.4
471.5
239.5
277.4
289.7
305.8
333.7
Intensity, cps
m/z, amu
-EMS: 7.195 to 7.395 min from Sample 4 (Mouse_Hep_120min_NoRAM) of Neg_EMS.wiff (Turbo Spray), subtracted (6.980 to 7.123 ...
Max. 1.4e5 cps.
271.1
447.1
469.1
112.9
269.0
567.1
Intensity, cps
m/z, amu

B

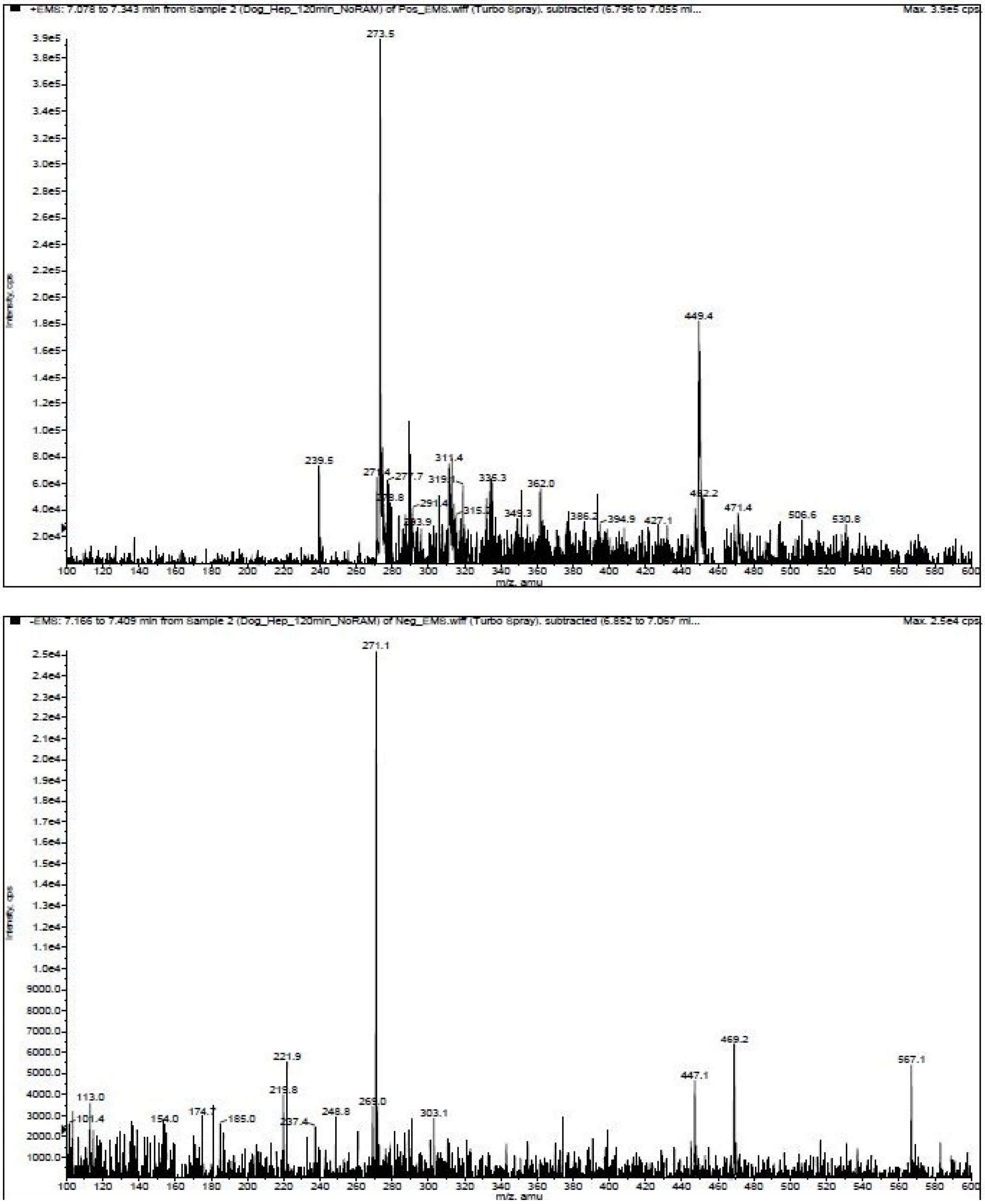
+EMS: 7.078 to 7.343 min from Sample 2 (Dog_Hep_120min_NoRAM) of Pos_EMS.wiff (Turbo Spray), subtracted (6.796 to 7.055 mi...
Max. 3.9e5 cps.
Intensity, cps
m/z, amu
273.5
449.4
239.5
311.4
336.3
362.0
277.7
471.4
506.6
530.8
427.1
394.9
-EMS: 7.166 to 7.409 min from Sample 2 (Dog_Hep_120min_NoRAM) of Neg_EMS.wiff (Turbo Spray), subtracted (6.852 to 7.067 mi...
Max. 2.5e4 cps.
Intensity, cps
m/z, amu
271.1
469.2
567.1
447.1
221.9
113.0
154.0
185.0
248.8
303.1

C

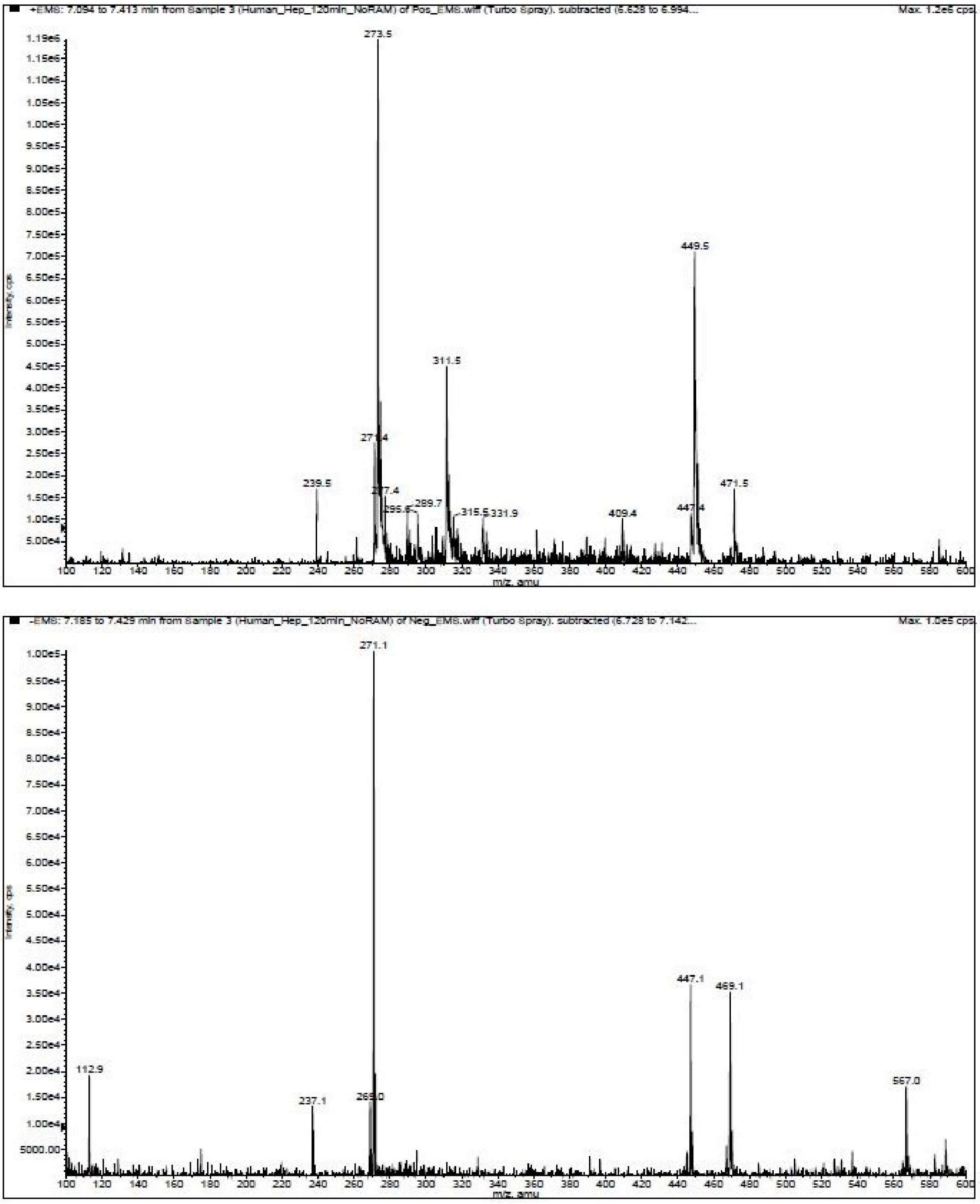

Figure S6. EMS Spectra of genistein 7-*O*-glucuronide in hepatocyte extract 2 h post addition of 10 μM [14C] genistein from (**A**) mouse, (**B**) dog, and (**C**) human; electrospray positive ion (top) and negative ion mode (bottom) for each molecular species. HPLC/RAD chromatograms generated from the hepatocyte samples showed the presence of up to 2 metabolites in addition to [14C] genistein. Representative 2-h incubation samples from mouse, dog, and human hepatocytes were used for metabolite identification. Metabolites were identified by comparison of their mass spectral fragmentation patterns with those of [14C] genistein. Mass spectra were obtained under positive ion and negative ion mode with an API3200 mass spectrometer in enhanced mass spectrum (EMS) mode to obtain molecular weight data.

Free genistein

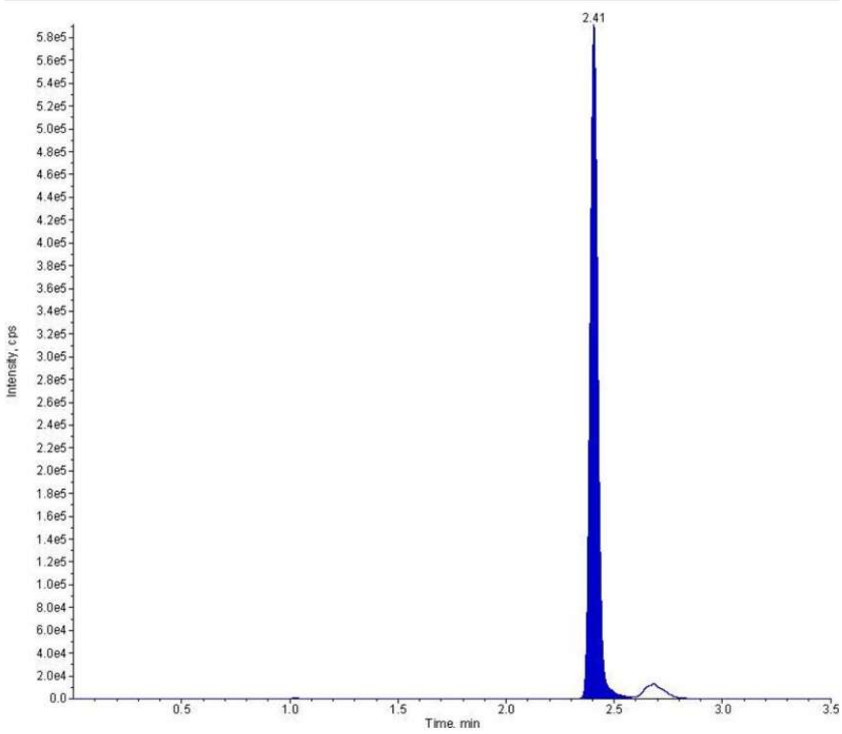

Total genistein

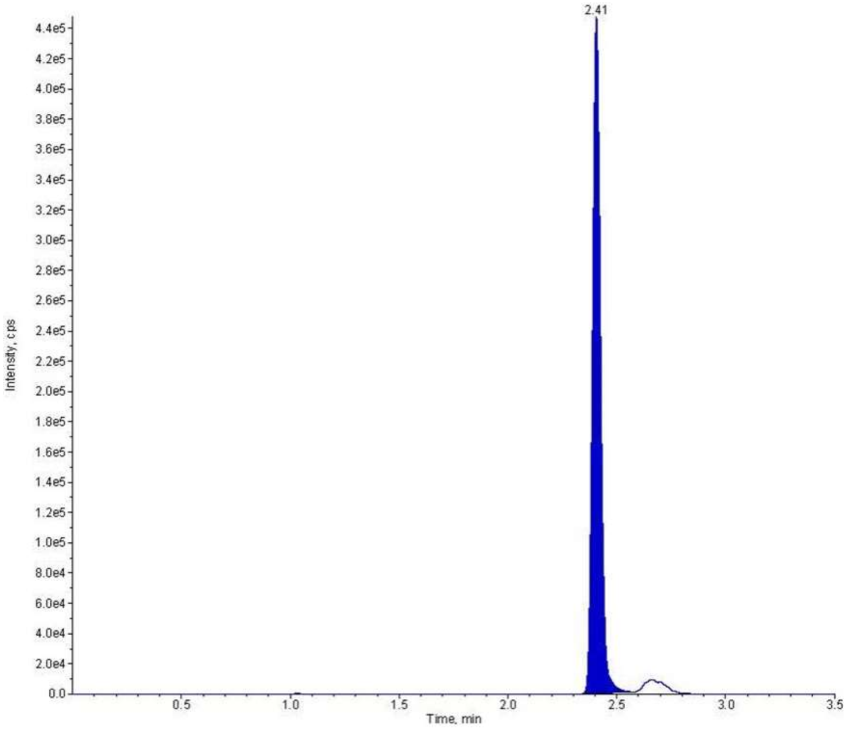

Figure S7. The extracted ion chromatogram of genistein (m/z = 273) at 1 ngm/mL. The data were acquired on a 3200 triple quadruple mass spectrometer (Sciex). Technical replicates showed an excellent reproducibility with respect to compound purity and chromatographic elution time (RT).
